# Mechanism of rotenone binding to respiratory complex I depends on ligand flexibility

**DOI:** 10.1038/s41598-023-33333-6

**Published:** 2023-04-25

**Authors:** Caroline S. Pereira, Murilo H. Teixeira, David A. Russell, Judy Hirst, Guilherme M. Arantes

**Affiliations:** 1 Department of Biochemistry, Instituto de Química, Universidade de São Paulo, Av. Prof. Lineu Prestes 748, São Paulo, SP 05508-900 Brazil; 2 MRC Mitochondrial Biology Unit, University of Cambridge, Cambridge Biomedical Campus, Hills Road, Cambridge, CB2 0XY UK; 3 Department of Chemistry, University of Cambridge, Lensfield Road, Cambridge, CB2 1EW UK

**Keywords:** Computational biophysics, Mitochondrial proteins, Oxidoreductases, Bioenergetics

## Abstract

Respiratory complex I is a major cellular energy transducer located in the inner mitochondrial membrane. Its inhibition by rotenone, a natural isoflavonoid, has been used for centuries by indigenous peoples to aid in fishing and, more recently, as a broad-spectrum pesticide or even a possible anticancer therapeutic. Unraveling the molecular mechanism of rotenone action will help to design tuned derivatives and to understand the still mysterious catalytic mechanism of complex I. Although composed of five fused rings, rotenone is a flexible molecule and populates two conformers, bent and straight. Here, a rotenone derivative locked in the straight form was synthesized and found to inhibit complex I with 600-fold less potency than natural rotenone. Large-scale molecular dynamics and free energy simulations of the pathway for ligand binding to complex I show that rotenone is more stable in the bent conformer, either free in the membrane or bound to the redox active site in the substrate-binding Q-channel. However, the straight conformer is necessary for passage from the membrane through the narrow entrance of the channel. The less potent inhibition of the synthesized derivative is therefore due to its lack of internal flexibility, and interconversion between bent and straight forms is required to enable efficient kinetics and high stability for rotenone binding. The ligand also induces reconfiguration of protein loops and side-chains inside the Q-channel similar to structural changes that occur in the open to closed conformational transition of complex I. Detailed understanding of ligand flexibility and interactions that determine rotenone binding may now be exploited to tune the properties of synthetic derivatives for specific applications.

## Introduction

Respiratory complex I is a fundamental oxidoreductase enzyme that generates much of the electrochemical gradient used to power oxidative phosphorylation in mitochondria. It catalyses electron transfer from reduced nicotinamide adenine dinucleotide (NADH) to ubiquinone (Q) coupled to transmembrane proton pumping^1,2^. The process is fully reversible and may also produce reactive oxygen species that damage cells and contribute to ischemia-reperfusion injury, neurodegenerative pathologies and ageing^3–5^.

**Figure 1:**
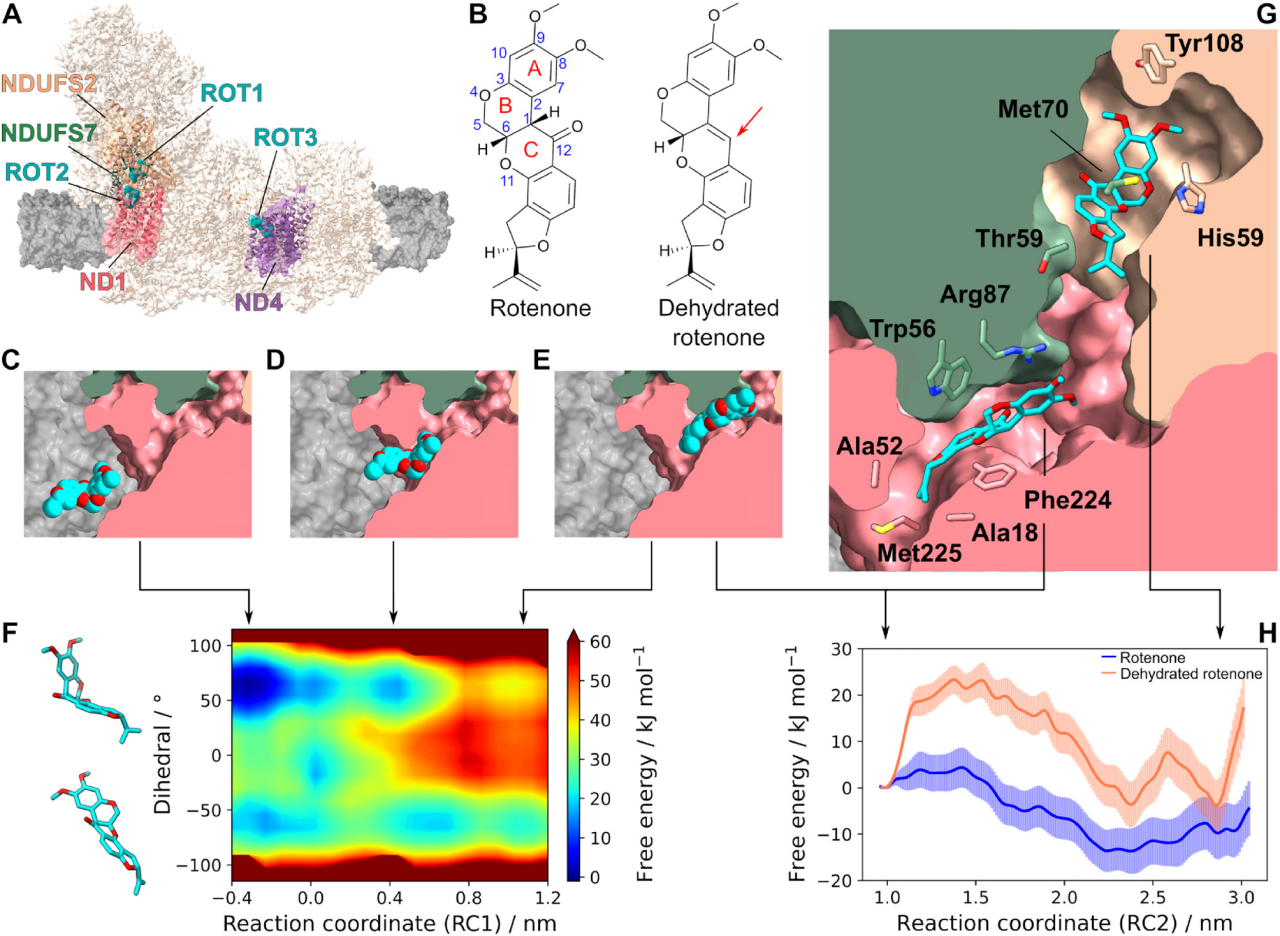
Mechanism of rotenone binding into respiratory complex I. (**A**) Structure of complex I is shown with subunits ND1, ND4, NDUFS2 and NDUFS7 colored respectively in pink, purple, peach and green, with the membrane in gray. Three rotenone binding modes (ROT1, ROT2 and ROT3) observed in cryoEM models^8,9^ are displayed in cyan spheres. (**B**) Chemical structures of rotenone (left) and dehydrated rotenone (right), with a red arrow indicating C1$=$C12 double bonding. Rotenone is shown in cyan (carbon) and red (oxygen) spheres on the (**C**) membrane, (**D**) narrow entrance and (**E**) ROT2 binding mode along the entry process. (**F**) Two-dimensional free energy profile for the entry process (corresponding to panels **C** $\rightarrow$ **D** $\rightarrow$ **E**). Rotenone structures in bent (dihedral $\chi _2 \approx +65^{\circ }$) and straight ($\chi _2 \approx -65^{\circ }$) conformations are shown on the left. (**G**) The Q-channel, with rotenone in binding modes ROT2 and ROT1. Two molecules are displayed here for visualisation, but only one was present during simulations. (**H**) Free energy profile for ligand transit from channel central region (ROT2) to Q-redox site (ROT1) for rotenone (blue) and dehydrated rotenone (orange). Uncertainties are shown in colored shadows. Arrows from panels (**C**–**E** and **G** point to the corresponding reaction coordinate in panels (**F**) (RC1) and (**H**) (RC2).

Mammalian complex I has 45 subunits arranged in an L-shaped structure (Fig. 1A), divided into a transmembrane domain and a hydrophilic arm. The natural substrate Q$_{10}$ (ubiquinone with a tail of 10 isoprenoid units) binds in a narrow, 35 Å-long cavity formed by subunits ND1, NDUFS2 and NDUFS7 at the interface of the two domains, connecting the membrane to the redox-active iron-sulfur (FeS) clusters^6–10^. This so-called Q-channel has three distinct regions (Fig. 1G): the hydrophobic and narrow channel entrance (or “doorway”) between the membrane and residues ND1 Ala18, Ala52 and Phe220 (numbering from complex I of *Mus musculus* is used throughout), the hydrophilic and highly hydrated central region between NDUFS7 Trp56, Arg87 and ND1 Arg274, and the amphipatic redox active site from NDUFS7 Met70 and up to NDUFS2 His59, Tyr108 and FeS cluster N2. The substrate Q-headgroup binds to the hydrophilic central region and to the redox active site (Fig. 1G)^6,11–13^. The binding mode(s) and exact contacts adopted by the Q-headgroup are variable and depend on the enzyme conformational state, Q-tail length and catalytic conditions^8–10^.

Two major conformational states of mammalian complex I have been structurally characterized by cryo-electron microscopy (cryoEM), differentiated by domain reorientations and reordering of loops around the Q-channel: a “closed” state with a tight and sealed channel, and an “open” state with more relaxed and disordered loops^8,14,15^. Their interpretation as either on-pathway catalytic intermediates^8,16^ or off-pathway resting states [the “ready-to-go” active resting state (closed) or pronounced “deactive” resting state (open) observed for mammalian complex I]^10,17–19^ is still a matter of debate^15^. Yet, it is agreed that closed enzyme conformations are catalytically relevant and, therefore, the closed form is the focus of this study.

A plethora of small molecules with diverse composition and physico-chemical properties inhibit the activity of complex I^20^. Recent structural data mainly from cryoEM have shown that many of these molecules bind into the amphipathic Q-channel^7,9,21–25^. For instance, short-tail substrate analogs such as piericidin A, a piridinone with antibiotic activity, and 2-decyl-4-quinazolinylamine respectively bind to the Q-redox site with poses similar to the substrate^7,10,22^ or slightly displaced towards NDUFS2 His59^21^. Acetogenins, amphiphilic natural products containing a $\gamma$-lactone linked to a bis-tetrahydrofuran group by an aliphatic chain, bind with these hydrophilic moieties respectively in the Q-redox site and central channel region^24^. Compound IACS-2858, a derivative of IACS-010759 which is under clinical trials as an anticancer agent, binds with its oxadiazole and pyridone moieties in the hydrophilic central region and competitively inhibits complex I by a “cork-in-bottle” mechanism^23^. The biguanide IM1761092, a more hydrophobic and potent derivative of the widely used metformin antidiabetic drug, also binds in the Q-channel central region^25^. Even detergent molecules used for protein purification, such as *n*-dodecyl $\beta$-D-maltoside (DDM), have been observed with their polar heads in the hydrophilic central region^26^.

Rotenone is one of the most widely used and most potent inhibitors of complex I (Fig. 1B)^27^. This lipophilic natural product has been used for centuries by indigenous peoples in South America to aid in catching fish and, more recently, broadly employed as a pesticide^28^ or a candidate anticancer lead compound because it selectively inhibits proliferation of neoplastic cells^29,30^. The rotenone molecule contains five fused rings divided in two nearly flat regions (rings A+B and rings C+D+E). X-ray crystallography of pure rotenone shows two conformations, bent and straight (Fig. 1F), depending on the orientation of the flat regions about the B/C ring junction^31,32^. CryoEM structures have identified three rotenone binding-modes in mammalian complex I: ROT1, ROT2 and ROT3 (Fig. 1A). ROT1 overlaps with the Q-redox site^8,9^, ROT2 occupies the Q-channel central and entrance regions^8^, and ROT3 is located in the ND4 subunit^8^. Rotenone binds in the ROT1 mode in both the closed and open conformational states, but has been observed in the ROT2 and ROT3 modes only in open states. Strikingly, rotenone binds in the bent and straight conformers in the ROT1 and ROT2 modes^8^, respectively (Fig. 1G). These observations, combined to an early suggestion that the bent form is necessary for binding^33^, raise the possibility of a role for rotenone intramolecular flexibility for binding into complex I.

Here, a dehydrated rotenone derivative (Fig. 1B) locked in the straight conformation was synthesized. This molecule still inhibits complex I catalysis but in concentrations much higher than rotenone. To understand the lower potency of this derived inhibitor, molecular dynamics (MD) and free energy simulations with enhanced sampling were used to probe the complete mechanism of rotenone binding into the narrow Q-channel of complex I. Results indicate that rotenone binds to the Q-redox site (ROT1) with higher stability in the bent form. But, efficient kinetics for passage through the narrow entrance and inside the channel relies on interconversion between bent and straight ligand conformations.

## Materials and methods

### Experimental section

Rotenone was obtained from Molekula Fine Chemicals (Darlington, UK, 90-95%) and was crystallized from EtOH three times^30^. Dehydrated rotenone was prepared from rotenone in two steps according to the method of Büchi et al.^34^, using acetyl chloride as the dehydrating agent in place of phosphorous oxychloride.

Inhibitor IC$_{50}$ values were measured from catalytic activity measurements on complex I in *Bos taurus* (bovine) heart mitochondrial membranes as described previously^30^. Normalized kinetic data were fit in Prism version 7.0e using a log(inhibitor) $\times$ normalized response fit with variable slope and presented with 95% confidence intervals.

### Set-up of protein model for molecular dynamics simulations

The simulation model employed here is based on complex I from *Mus musculus* in the closed state (PDB 6ZR2)^22^. It was assumed that the complete rotenone binding process occurs in this conformational state. The model is the same as used recently to simulate Q$_{10}$ substrate binding^10^. Further details on model set-up are described in the Supporting Information (SI). All cofactors and post-translational modifications present in the PDB model were included. High confidence phospholipids were retained. Protonation states of side-chains were adjusted to neutral pH, but NDUFS2 His59 was double-protonated (Hsp)^35^. The FeS cluster N2 was simulated in the reduced state^36^.

The protein complex was embedded in a solvated bilayer mimicking the inner mitochondrial membrane with a composition of 47% DLPC (1,2-dilinoleoyl-sn-glycero-3-phosphatidylcholine), 38% DLPE (1,2-dilinoleoyl-sn-glycero-3-phosphatidylethanolamine), 12% CDL (1$^{\prime }$-3$^{\prime }$-bis[1,2-dilinoleoyl-sn-glycero-3-phospho]-sn-glycerol, cardiolipin dianion), and 3% of ubiquinone Q$_{10}$. This simulation model was relaxed and equilibrated during molecular dynamics (MD) simulations of 360 ns^10^. We note as a correction to our previous work^10^ that force constants are in units of kJ mol$^{-1}$ nm$^{-2}$.

**Figure 2:**
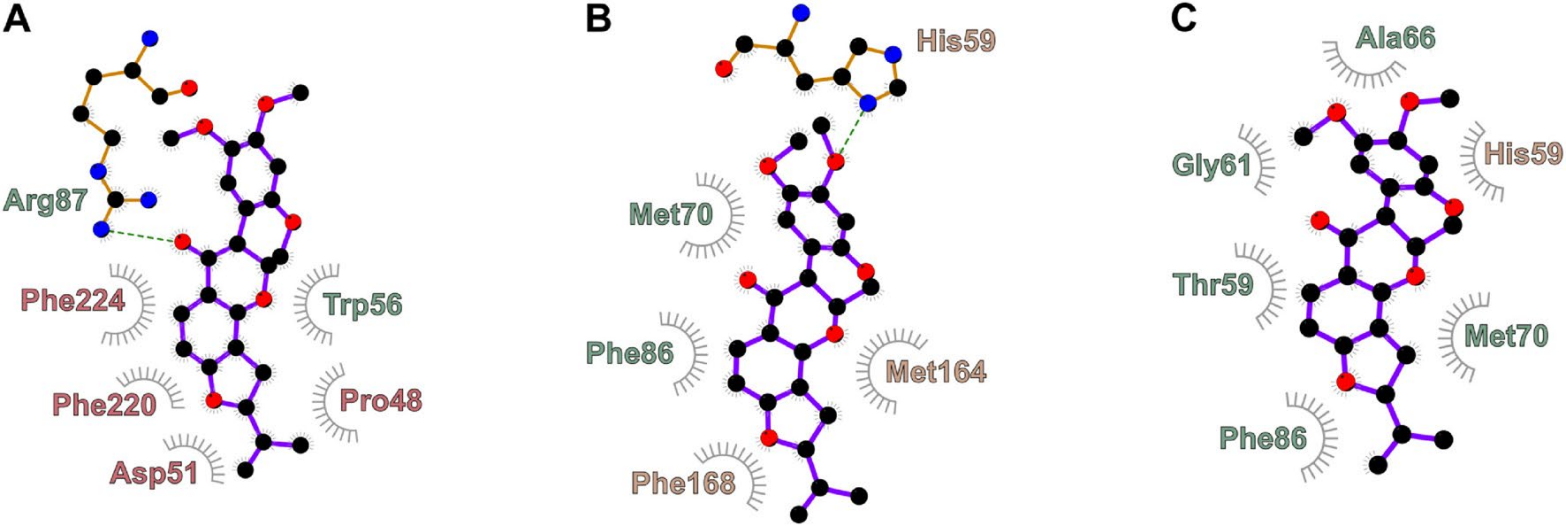
Rotenone contacts to residues in complex I with high interaction energies at binding modes (**A**) ROT2, (**B**) pre-redox and (**C**) ROT1. Residue names are colored as their subunits in Fig. 1.

An unbound Q-channel was produced by erasing the bound Q from our previously equilibrated model^10^ and one rotenone was added manually to each binding mode ROT1, ROT2 and ROT3, accordingly to previous cryoEM models (PDB 6ZKM)^8^. Water molecules were added in the Q-channel and adjusted accordingly to previous experimental structures (PDB 6YJ4)^26^. The ND4 backbone near ROT3 was manually adjusted to fit the geometry observed in the cryoEM structure (PDB 6ZKM). No adjustments were used for protein centers near ROT1 and ROT2 sites, so their geometry corresponds to the equilibrated model^10^ of the closed conformation^22^. The resulting simulation model contained 862,234 atoms. Single rotenone models used for canonical and enhanced sampling MD simulations were obtained by erasing the other two rotenones and replacing by water molecules, with additional 200 ns of MD for equilibration. Simulations of dehydrated rotenone started by replacing the ligand in ROT2 and were further equilibrated by a MD run of 70 ns. Pair interaction energies were calculated for residues within 0.5 nm of rotenone in the Q-channel using the last 50 ns of MD simulations with rotenone at the binding modes identified. Residues with average pair energy with modulus greater than 10 kJ mol$^{-1}$ were considered in Fig. 2.

All simulations were conducted with GROMACS (version 2020.3)^37^ at constant temperature of 310 K, pressure of 1 atm and a time step of 2 fs. Long-range electrostatics were treated with the Particle Mesh Ewald method^38^. Interactions of protein, cofactors, lipids and ions were described with the all-atom CHARMM36m force field (charmm36-mar2019.ff)^39^. Water was represented by the standard TIP3P model^40^. FeS centers were described using the Chang & Kim parameters^41^ with corrections^42^. Our calibrated parameters were used for Q$_{10}$^43,44^. The force field for rotenone and its derivative was initially generated with CGenFF^45^. Energetics for torsion around dihedrals C2-C1-C6-O7 and C1-C6-C5-O4 (Fig. 1B), herein respectively dihedrals $\chi _1$ and $\chi _2$, were checked and fitted to B3LYP/6-31+G** torsion profiles in the gas-phase^46–48^. These rotenone parameters are included in Tables S1 and S2 (SI) and complete force-field files are available online^49^.

### Reaction coordinates and free-energy calculations

The complete rotenone binding process, with ligand entry from the membrane phase into the Q-channel central region (ROT2) and up to the Q-redox position (ROT1), had to be described in two separate sets of simulations, each employing a different definition of reaction coordinate (RC). Rotenone entry from the membrane to ROT2 was described by RC1, corresponding to the Z-component of the distance between the center of mass (COM) of C2, C3, C7, C8, C9, C10 in rotenone and the COM of C$\alpha$ from Ala18, Ala52 and Met225 in the NDUFS7 subunit (Fig. 1G). These residues form the narrow entrance of the Q-channel in contact with the membrane. Rotenone transit inside the Q-channel (ROT2$\rightarrow$ROT1) was described by RC2, corresponding to 4 nm minus the distance between the COM of the same rotenone atoms as in RC1 to the COM of C$\alpha$ from the following residues located near the FeS N2 cluster: Leu106, Asp107, Tyr108, Val109, Ser110, Met189, His190, Ala191, Val424, Gly426 and Glu427 in the NDUFS2 subunit. These two RC were defined so that binding proceeds from the membrane into the Q-channel, from lower to higher RC values.

Dihedrals $\chi _1$ and $\chi _2$ were used to describe the rotenone internal bent $\rightleftharpoons$ straight transition. For instance, $\chi _2 \approx +65^{\circ }$ in the bent conformer and $\chi _2 \approx -65^{\circ }$ in the straight form. To increase sampling, well-tempered metadynamics^50^ was activated in $\chi _1$ and $\chi _2$ with Gaussians deposited every 250 time steps (0.5 ps), at initial height of 1.2 kJ mol$^{-1}$, widths of 0.2 and a bias factor of 15. Umbrella sampling was performed along the range RC1 = [− 0.4, 1.2] nm in 6 equally spaced windows plus 3 extra windows to increase RC1 overlap, centered at RC1 = {0.18, 0.72, 0.82} nm. RC2 was sampled along the range RC2 = [0.9, 3.2] nm, in 24 windows equally spaced, with $k_{umb} =$ 1000 kJ mol$^{-1}$ nm$^{-2}$. In both simulation sets, each window was sampled for 170 ns. Umbrella and metadynamics potentials were included with the PLUMED plugin (version 2.6.1)^51^. The metadynamics bias was removed by re-weighting the distribution of RC in each window and free energy profiles were generated using WHAM^52^, with statistical uncertainties estimated by bootstrap analysis with 95% confidence intervals. Steered MDs were performed to generate configurations for umbrella sampling with a moving harmonic restraint of $k_{rest} =$ 1000 kJ mol$^{-1}$ nm$^{-2}$ along the reaction coordinate from ROT2 to ROT1 in a total duration of 5 ns and from membrane to ROT2 in 1.5 ns.

## Results and discussion

The NADH:O2 oxidoreductase activity of respiratory complex I from bovine heart mitochondria was measured and half-maximal inhibitory concentrations (IC$_{50}$ values) of rotenone and dehydrated rotenone were determined as 6.9 nM^30^ and 4 $\upmu$M, respectively^53^.

Both ligands have comparable hydrophobicities (estimated using MarvinSketch^54^) so they are expected to partition similarly in the membrane phase. However, dehydrated rotenone is locked in the straight conformation due to the double bond introduced in C1$=$C12 (Fig. 1B). We conjectured that the 600-fold difference in inhibition potency is caused by a lower stability of the straight conformer in the Q-redox site. To understand the binding process in detail and test this proposal, molecular dynamics and free-energy simulations were used to investigate the complete binding pathway of rotenone from the membrane into the Q-channel.

In the first set of simulations, the relative position of rotenone in the Q-channel (Fig. 1C–E) is described by reaction coordinate RC1 (defined in “Materials and methods”). The ligand is out in the membrane when RC1 < 0 nm and progressively enters the channel as RC1 increases. RC1$=$1.0 nm corresponds to rotenone bound at experimental mode ROT2. Dihedral $\chi _2$ is used to describe the ligand internal conformation. The bent form has $\chi _2 \approx +65^{\circ }$ and the straight form has $\chi _2 \approx -65^{\circ }$. The free energy surface computed for rotenone entry is shown in Fig. 1F.

In the membrane and not in direct contact with the protein (Fig. 1C,F with RC1$\approx -$0.3 nm), rotenone is 12 kJ/mol more stable in the bent conformer. This corresponds to a population ratio of 100:1 between the two forms. Their interconversion has a barrier of 31 kJ/mol, which is 12-fold the thermal energy at T = 310 K ($k_B$T, where $k_B$ is the Boltzmann constant). These values are an intrinsic property of rotenone and are retained to within ±3 kJ/mol when rotenone is embedded in a membrane with the same lipid composition but free of any protein (Fig. S1A) or in the water phase (not shown). Thus, the relative stability and interconversion rate of the conformers do not depend on the environment polarity.

Embedded in the membrane, rotenone ring A localizes close to glycerol groups of phospholipids (Fig. S1B), similar to the Q-headgroup position of ubiquinone in membrane models^43,44^. Rotenone orients almost normal to the membrane plane, with rings D and E buried in the hydrocarbonic region. This location allows efficient lateral diffusion and interaction of rotenone ring A with ND1 residues Ala52 and Met225 exposed to the membrane (Fig. 1C,G).

As soon as rotenone ring A binds in the narrow channel entrance (RC1 = 0.0 nm, Fig. 1F), the interconversion barrier decreases by about 10 kJ/mol, speeding up conformational exchange by 50-fold. Thus, at this ligand position, the spatially limited Q-channel *catalyses* the rotenone intramolecular conversion. Further ligand entry (0.4 $\le$ RC1 < 1.0 nm) destabilizes the bent conformation by as much as 50 kJ/mol, reversing the population ratio up to 1:10,000 for bent and straight forms.

The free energy barrier for rotenone entry into the channel is 26 kJ/mol ($\sim$10 k$_B$T) at RC1 = 0.85 nm in the straight conformation (dihedral $\chi _2 = -$65$^{\circ }$). Passage through this point is rate-limiting for the complete rotenone binding process from membrane to the Q-redox site (ROT1). The barrier found for entry in the bent conformation is much higher and surpasses 50 kJ/mol, corresponding to a rate 4 orders of magnitude slower, and precluding efficient ligand binding into complex I for the bent form. Thus, rotenone internal flexibility and the transition from the bent (intrinsically more stable in the free ligand) to the straight conformer allows rapid entry through the narrow Q-channel and an efficient binding kinetics (high $k_{on}$).

It has been proposed that passage of the ubiquinone substrate through the narrow Q-channel entrance and the hydrophobic bottleneck near ND1 Ala18, Ala52 and Met225 (Fig. 1G), is not possible in closed enzyme conformations^8,55,56^. This suggestion is in striking contrast to our previous free-energy simulations of ubiquinone (Q$_{10}$ and Q$_2$) binding that show passage through the bottleneck in *T. thermophilus* complex I with a tight and sealed Q-channel (closed-like conformer) is feasible, with low free energy cost and without significant protein deformation^12^. The ubiquinone headgroup has similar composition and molecular volume to rotenone ring A (Fig. 1B). Simulations presented here clearly show that passage of rotenone in the straight form through the bottleneck is also energetically feasible for the closed state of the Q-channel in mammalian complex I (Fig. S2).

Rotenone reaches a free energy minimum at RC1 = 1.0 nm in the straight conformation. At this point, the ligand hydrogen-bonds to NDUFS7 Arg87 with its carbonyl (C12=O) and to internal waters with its methoxy oxygens. Hydrophobic contacts with ND1 Phe224 and NDUFS7 Trp56 are also formed (Figs. 1G, 2A). These contacts, the binding mode and the ligand internal conformation match those observed for rotenone bound at ROT2 in the cryoEM model of complex I in open state (PDBs 6ZKL and 6ZKM)^8^. Moreover, this corresponds to the binding site closest to the Q-channel central region also observed for the substrate Q-headgroup^6,12^ and other amphiphilic molecules^23,25,26^.

In the second set of simulations, the relative position of the ligand in the Q-channel is described by reaction coordinate RC2. It is defined so that both RC1 and RC2 are equal to 1.0 nm when the ligand is bound at ROT2. For rotenone, the free energy profile in Fig. 1H represents the free energy pathway along RC2 integrating over dihedral coordinates that describe its internal flexibility ($\chi _1$ and $\chi _2$, see Methods). This allows plotting together and comparing profiles for both rotenone and its dehydrated derivative.

At the central region, the Q-channel has a kink at 1.2 < RC2 < 1.5 nm and near NDUFS7 Arg87. Rotenone flexibility is once again important for kinetically efficient passage through this region, but now with the straight$\rightarrow$bent transition. The channel cavity after the kink is broader and able to accommodate the rotenone molecule in the intrinsically more stable bent conformation (Fig. 1G). Rotenone transit through this region shows a low barrier (5 kJ/mol, Fig. 1H), corresponding to simultaneous ligand progress inside the channel and transition to the bent conformation. This form is 5–10 kJ/mol more stable along the remaining Q-channel (RC2 > 2.0 nm), corresponding to a relative population 7- to 50-fold higher for the bent form than straight. No further intramolecular rotenone transitions are necessary to reach the Q-redox site.

Before ROT1, rotenone visits another free energy minimum at 2.2 < RC2 < 2.4 nm, herein denoted the “pre-redox” binding mode. This is the lowest minimum with rotenone inside the channel: it is 13 kJ/mol and 4 kJ/mol more stable than binding at ROT2 and ROT1 (RC2 = 2.8 nm), respectively. These free energy differences correspond to a ratio of 1:150:30 for the relative probability of occupation by a rotenone molecule in modes ROT2:pre-redox:ROT1.

At the pre-redox binding mode, rotenone hydrogen bonds with the charged side-chain of NDUFS2 His59 via its methoxy oxygens (Fig. 2B). This contact and binding mode for the rotenone ring A are comparable to those found previously for the Q-headgroup in the pre-reactive site during simulation of ubiquinone (Q$_{10}$ and Q$_{2}$) binding into *T. thermophilus* complex I^12^ and observed in the cryoEM model for Q$_{10}$ bound into mammalian complex I^10^.

In the shallow free energy minimum found at site ROT1 (RC2 = 2.8 nm), rotenone makes strong contacts with NDUFS7 Met70, Thr59 and NDUFS2 His59, and hydrogen bonds to internal water with its methoxy oxygens (Figs. 1G and 2C). These contacts, the binding mode and the ligand conformation match those observed in cryoEM structures of rotenone bound at the ROT1 mode for complex I in the closed state, in enzyme preparations with (PDBs 6ZKK and 7V33) or without NADH (PDB 7V31)^8,9^. The root mean square deviation (RMSD) for the position of rotenone heavy-atoms between these last two cryoEM models after alignment of NDUFS2 and NDUFS7 backbones is only 0.03 nm, with the ligand in bent conformation in both models.

**Figure 3:**
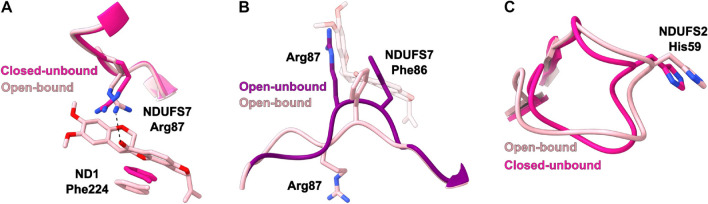
Rotenone-induced conformational changes in (**A**) ND1 Phe224 and NDUFS7 Arg87 side-chains near the ROT2 binding mode, (**B**) NDUFS7 Arg87 loop near the pre-redox mode, and (**C**) NDUFS2 $\beta$1-$\beta$2 loop near the ROT1 mode. Panels (**A**) and (**B**) show a rotenone obtained from simulations in the ROT2 and pre-redox modes, respectively. Closed-unbound corresponds to PDB 6ZKG^8^ and is equivalent to other closed cryoEM models without ligands inside the Q-channel, such as PDB 6ZR2^22^ used to start the simulations here. Open-unbound corresponds to PDB 6ZKS^8^, which has a disordered NDUFS2 $\beta$1–$\beta$2 loop similar to other open cryoEM models^15^. Open-bound corresponds to PDB 6ZKL^8^, with rotenone bound in both ROT1 and ROT2 modes.

Rotenone is also bound to ROT1 in open complex I cryoEM models (PDB 6ZKL and 6ZKM)^8^, without significant changes in its position or direct contacts to the protein (RMSD = 0.02 nm to rotenone in PDB 6ZKK). Notably, the NDUFS2 $\beta$1–$\beta$2 loop is ordered in these open-bound cryoEM models, with conformation equivalent to closed-unbound models and in contrast to open-unbound models, when the loop is disordered (Fig. 3C)^8,15^. Besides reshaping of this NDUFS2 loop and the NDUFS7 Arg87 loop (see below), all other structural hallmarks of the open conformation^15^ are retained in open-bound structures. Thus, binding of inhibitors such as rotenone can induce local ordering around the Q-redox site, which may lead to partial reactivation of the enzyme^57^. It may be concluded that ROT1 is a stable rotenone binding mode, in both closed and open conformational states^8,57^, and independent of NADH content (consequently, N2 cluster redox state) in the closed state^9^.

A rotenone molecule bound to ROT2 was only built in open state cryoEM models (PDBs 6ZKL and 6ZKM)^8^. The NDUFS7 Arg87 loop in these open-bound models is also reshaped to a closed conformation^8,15^, disrupting a cation-$\pi$ stacking^58^ found between Arg87 and Phe86 in open-unbound models and placing Arg87 towards the Q-channel entrance (Fig. 3B).

In our initial simulation model (PDB 6ZR2)^22^, positions of side-chains that perform strong contacts with rotenone in ROT2 (Fig. 2A) are similar to *apo*-ROT2 *holo*-ROT1 closed model (PDB 6ZKK)^8^ and to other closed-unbound forms^15^, except for side-chains of Arg87, which is rotated away and not in position to hydrogen-bond with rotenone C12=O carbonyl, and of ND1 Phe224, that protrudes 0.2 nm into the site, slightly overlapping with the position of rotenone rings B and C in ROT2 (Fig. 3A). During simulations, these side-chains easily convert to configurations found in open-bound cryoEM models as soon as rotenone occupies the Q-channel central region, showing that structural changes in side-chains with strong interactions with rotenone can be induced by ligand binding.

Simulations also indicate that binding in the ROT2 mode is 9–13 kJ/mol less stable than binding to the Q-redox site, corresponding to a 30- to 150-fold lower occupation for ROT2 in comparison to ROT1. This should hinder observation of densities and the assignment of a rotenone molecule bound to ROT2 in cryoEM models for the closed state. Nevertheless, densities were observed at the Q-channel central region of closed state maps for preparations containing rotenone, but they were modeled as the substrate Q-headgroup (PDB 7V31 and 7V33)^9^. For the open state, rotenone binding to ROT2 may be stabilized through long-range or indirect interactions (beyond those probed in the closed model here and shown in Fig. 2) that allow its clear observation in cryoEM maps.

Although the pre-redox site is not confirmed by current rotenone-bound structures^8,9^, a clear and continuous density is observed near the pre-redox site (Fig. S3) for the cryoEM map in the closed state of *Sus scrofa* complex I prepared with NADH and rotenone (PDB 7V33 and EMD-31651)^9^. This density can approximately fit a rotenone molecule and can not be assigned to the protein chain. A similar density at the pre-redox site was not observed for other cryoEM maps of rotenone-bound complex I. But, two molecules had to be concomitantly modeled to fit the density observed inside the Q-channel, either two rotenones (PDBs 6ZKL and 6ZKM^8^) or one rotenone (at ROT1) and one substrate Q (at the channel central region, PDB 7V31)^9^. For the open state, it is probably harder to observe rotenone binding at the pre-redox mode because it overlaps with the NDUFS7 Arg87-Phe86 cation-$\pi$ contact formed in the open-unbound conformation (Fig. 3B).

One of the cryoEM models of complex I in the open state (PDB 6ZKM)^8^ indicated rotenone binding to a cavity exposed to the membrane in the ND4 subunit (ROT3, Fig. 1A). However, the ligand built in this model is not rotenone but an epimer, with an inverted stereochemical configuration at C6 (compare Figs. 1B and S4). Another recent cryoEM model of complex I in the “slack” conformational state (also called state 3, PDB 7QSO)^10^ indicated Q$_{10}$ binding to the same ND4 site, with the Q-headgroup overlapping the position observed for rotenone ring A^8^. After adjusting the ND4 backbone around this cavity (ROT3) to the cryoEM model and using the correct rotenone stereoisomer, MD simulations and a linear interaction energy (LIE) analysis^59,60^ yielded a difference in relative free energy $\Delta \Delta$G$_{LIE}$ = +10 kJ/mol for binding to ROT3 in relation to ROT2. This unfavorable energetics corresponds to a 50-fold lower occupation of ROT3 in relation to the already less populated ROT2, and suggests weak rotenone binding to the ND4 cavity, only attainable in artificially high (*in vitro*) ligand concentrations. Yet, the inhibitory relevance of this ND4 site (ROT3) is further disputable as it has been observed only in prounced open states that are probably catalytically incompetent^15^.

Dehydrated rotenone (Fig. 1B) has little internal flexibility and is only able to populate the straight conformation. Thus, its entrance from the membrane to the Q-channel is similar to rotenone in the straight form, with a free energy profile following a line parallel to the x-axis with dihedral $\chi _2 = -65^{\circ }$ in the surface of Fig. 1F. However, transit inside the Q-channel is different. Moving dehydrated rotenone further into the channel from the ROT2 position requires it to cross a steep free energy barrier of 23 kJ/mol between 1.2 < RC2 < 1.8 nm (Fig. 1H). This barrier is 18 kJ/mol higher for dehydrated rotenone than for rotenone, corresponding to $\sim$ 1000-fold slower transit rate. This is because the kink in the Q-channel near NDUFS7 Arg87 restricts ligand movement and hinders transit for the rigid derivative.

Dehydrated rotenone also binds in the pre-redox and ROT1 modes with a clear free energy barrier in-between, but with a computed stability in the Q-redox site $\Delta \Delta$G$_{comp}$ = 10 kJ/mol lower than rotenone (Fig. 1H). The difference in binding free energy in the ROT2 mode between rotenone in its straight form and its derivative was not computed here but assumed to be zero. Both profiles in Fig. 1H were set by construction to the same free energy value at ROT2 (RC2 = 1.0 nm). However, dehydrated rotenone lacks a carbonyl at C12 and is not able to hydrogen-bond with NDUFS7 Arg87, suggesting it will have a lower affinity to ROT2 than rotenone. Consequently, the entire free energy profile for the dehydrated derivative (orange curve in Fig. 1H) should be shifted up in relation to the rotenone profile, increasing the difference in stability at ROT1 between the two ligands and approaching the difference estimated from the ratio of experimental IC$_{50}$s (see above), $\Delta \Delta$G$_{exp}$ = 16 kJ/mol. Therefore, the dehydrated derivative binds at the ROT2 and Q-redox sites with lower affinity than rotenone, which accounts for its 600-fold lower inhibition potency.

The lower inhibition reported for complex I from various species can be connected to mutations in residues identified here as strongly interacting with rotenone (Fig. 2). For instance, complex I from *E. coli* shows IC$_{50}$ values 2000-fold higher^61,62^ than the bovine enzyme. This can be traced to mutations at *E. coli* NDUFS7 Met70, Phe86, Thr59 and Ala66 near the Q-redox site. Complex I from *S. tuberosum* (potato), *T. brucei* and *P. denitrificans* are 200 to 1500-fold less sensitive to rotenone^23,61–63^. The three enzymes have ND1 Phe224 mutated to Tyr, Ser and Met, respectively. Complex I from *T. brucei* and *P. denitrificans* also have the NDUFS7 Phe86Pro mutation. Similarly, mutation Met70Cys in the NDUFS7 subunit of *Y. lipolytica* results in a rotenone-resistant enzyme^21^.

## Conclusions

Rotenone has been used for centuries as a selective and potent inhibitor of the respiratory complex I, and recently raised interest as a lead compound for developing cancer therapeutics^29,30^. The rotenone structure with five fused rings populates two conformers, bent and straight. Molecular dynamics and free energy simulations presented here indicate that interconversions between these two forms are necessary for rotenone binding in the Q-channel of complex I. The bent form is more stable, either when free in the membrane or when rotenone is bound in the Q-redox site. But, efficient kinetics for passage through the narrow entrance requires the straight conformation and transit through the kink near NDUFS7 Arg87 is faster when rotenone is in bent form.

The rotenone derivative synthesized here has 600-fold lower inhibition potency towards mammalian complex I because it has a lower affinity to the Q-redox site in comparison to natural rotenone. The derivative is locked in the straight form (due to the introduced C1$=$C12 double bond) and passes through the Q-channel kink with more difficulty, also resulting in slower binding to the Q-redox site.

Simulations reproduce both ROT1 and ROT2 experimental binding modes^8^, estimate their relative stabilities and indicate a new binding mode (pre-redox) that fits to unassigned density observed in a previous cryoEM map for the closed state^9^. Simulations also helped to analyze conformational changes induced by rotenone in loops and side-chains at the Q-channel that resemble some of the structural changes observed in transitions from open to closed states of complex I.

Internal flexibility in rotenone, and presumably other inhibitors^23^, is necessary for binding through the narrow Q-channel. This property may be exploited through synthetic efforts to selectively modulate the binding kinetics and stability of different conformations of rotenone derivatives. The complete mechanism of rotenone binding described here and the strong protein interactions observed in simulations may now be used to guide these efforts.

## Supplementary Information

Supplementary Information.

## Data Availability

Three snapshots from MD simulations containing subunits ND1, NDUFS2 and NDUFS7 and rotenone in binding modes ROT1, pre-redox and ROT2, and force-field files for rotenone and the dehydrated derivative in GROMACS format were uploaded online (DOI: 10.5281/zenodo.7781922)^49^. The complete datasets generated and/or analyzed during the current study are available from the corresponding author on reasonable request.

## References

[1] Hirst J (2013). Mitochondrial Complex I. Annu. Rev. Biochem..

[2] Parey K, Wirth C, Vonck J, Zickermann V (2020). Respiratory complex I - Structure, mechanism and evolution. Curr. Opin. Struct. Biol..

[3] Kussmaul L, Hirst J (2006). The mechanism of superoxide production by NADH: ubiquinone oxidoreductase (complex I) from bovine heart mitochondria. Proc. Natl. Acad. Sci. U.S.A..

[4] Murphy MP, Hartley RC (2018). Mitochondria as a therapeutic target for common pathologies. Nat. Rev. Drug Discov..

[5] Yin Z, Burger N, Kula-Alwar D, Aksentijević D, Bridges HR, Prag HA, Grba DN, Viscomi C, James AM, Mottahedin A, Krieg T, Murphy MP, Hirst J (2021). Structural basis for a complex I mutation that blocks pathological ROS production. Nat. Commun..

[6] Parey, K., Haapanen, O., Sharma, V., Köfeler, H., Züllig, T., Prinz, S., Siegmund, K., Wittig, I., Mills, D. J., Vonck, J., Kühlbrandt, W., Zickermann, V. High-resolution cryo-EM structures of respiratory complex I: Mechanism, assembly, and disease. *Sci. Adv.* **5** (2019) 10.1126/sciadv.aax9484 PMC6905873 31844670

[7] Gutiérrez-Fernández J, Kaszuba K, Minhas GS, Baradaran R, Tambalo M, Gallagher DT, Sazanov LA (2020). Key role of quinone in the mechanism of respiratory complex I. Nat. Commun..

[8] Kampjut, D., & Sazanov, L. A. The coupling mechanism of mammalian respiratory complex I. *Science* **370**, eabc4209 (2020). 10.1126/science.abc4209 32972993

[9] Gu J, Liu T, Guo R, Zhang L, Yang M (2022). The coupling mechanism of mammalian mitochondrial complex I. Nat. Struct. Mol. Biol..

[10] Chung I, Wright JJ, Bridges HR, Ivanov BS, Biner O, Pereira CS, Arantes GM, Hirst J (2022). Cryo-EM structures define ubiquinone-10 binding to mitochondrial complex I and conformational transitions accompanying Q-site occupancy. Nat. Commun..

[11] Warnau J, Sharma V, Gamiz-Hernandez AP, Luca AD, Haapanen O, Vattulainen I, Wikstrom M, Hummer G, Kaila VRI (2018). Redox-coupled quinone dynamics in the respiratory complex I. Proc. Natl. Acad. Sci. U.S.A..

[12] Teixeira MH, Arantes GM (2019). Balanced Internal Hydration Discriminates Substrate Binding to Respiratory Complex I. Biochim. Biophys. Acta.

[13] Haapanen O, Reidelbach M, Sharma V (2020). Coupling of quinone dynamics to proton pumping in respiratory complex I. Biochim. Biophys. Acta.

[14] Agip A-NA, Blaza JN, Bridges HR, Viscomi C, Rawson S, Muench SP, Hirst J (2018). Cryo-EM structures of complex I from mouse heart mitochondria in two biochemically defined states. Nat. Struct. Mol. Biol..

[15] Hirst J, Chung I, Grba DN, Wright JJ (2022). Making the leap from structure to mechanism: are the open states of mammalian complex I identified by cryoEM resting states or catalytic intermediates?. Curr. Opin. Struct. Biol..

[16] Kravchuk, V., Petrova, O., Kampjut, D., Wojciechowska-Bason, A., Breese, Z., Sazanov, L. A universal coupling mechanism of respiratory complex I. Nature **609**, 808–814. 10.1038/s41586-022-05199-7 36104567

[17] Kotlyar AB, Vinogradov AD (1990). Slow active/inactive transition of the mitochondrial NADH-ubiquinone reductase. Biochim. Biophys. Acta.

[18] Dröse S, Stepanova A, Galkin A (2016). Ischemic A/D transition of mitochondrial complex I and its role in ROS generation. Biochim. Biophys. Acta.

[19] Zhu J, Vinothkumar KR, Hirst J (2016). Structure of mammalian respiratory complex I. Nature.

[20] Murai M, Miyoshi H (2016). Current topics on inhibitors of respiratory complex I. Biochim. Biophys. Acta.

[21] Zickermann V, Wirth C, Nasiri H, Siegmund K, Schwalbe H, Hunte C, Brandt U (2015). Mechanistic insight from the crystal structure of mitochondrial complex I. Science.

[22] Bridges HR, Fedor JG, Blaza JN, Luca AD, Jussupow A, Jarman OD, Wright JJ, Agip A-NA, Gamiz-Hernandez AP, Roessler MM, Kaila VRI, Hirst J (2020). Structure of inhibitor-bound mammalian complex I. Nat. Commun..

[23] Chung, I., Serreli, R., Cross, J. B., Francesco, M. E. D., Marszalek, J. R., & Hirst, J. Cork-in-bottle mechanism of inhibitor binding to mammalian complex I. *Sci. Adv.* **7**, abg4000 (2021). 10.1126/sciadv.abg4000 PMC8121435 33990335

[24] Grba, D. N., Blaza, J. N., Bridges, H. R., Agip, A.-N. A., Yin, Z., Murai, M., Miyoshi, H., & Hirst, J. Cryo-electron microscopy reveals how acetogenins inhibit mitochondrial respiratory complex I. *J. Biol. Chem.* **298** (2022). 10.1016/j.jbc.2022.101602 PMC8861642 35063503

[25] Bridges HR, Blaza JN, Yin Z, Chung I, Pollak MN, Hirst J (2023). Structural basis of mammalian respiratory complex I inhibition by medicinal biguanides. Science.

[26] Grba DN, Hirst J (2020). Mitochondrial complex I structure reveals ordered water molecules for catalysis and proton translocation. Nat. Struct. Mol. Biol..

[27] Lümmen P (1998). Complex I inhibitors as insecticides and acaricides. Biochim. Biophys. Acta.

[28] Forge FBL, Haller HL, Smith LE (1933). The Determination of the Structure of Rotenone. Chem. Rev..

[29] Naguib A, Mathew G, Reczek CR, Watrud K, Ambrico A, Herzka T, Salas IC, Lee MF, El-Amine N, Zheng W, Francesco MED, Marszalek JR, Pappin DJ, Chandel NS, Trotman LC (2018). Mitochondrial Complex I Inhibitors Expose a Vulnerability for Selective Killing of Pten-Null Cells. Cell Rep..

[30] Russell DA, Bridges HR, Serreli R, Kidd SL, Mateu N, Osberger TJ, Sore HF, Hirst J, Spring DR (2020). Hydroxylated rotenoids selectively inhibit the proliferation of prostate cancer cells. J. Nat. Prod..

[31] Rossi M, Fule P, Taylor M (1988). Conformational flexibility in the molecular structure of rotenone, a naturally occurring insecticide. Bioorg. Chem..

[32] Fang N, Rowlands JC, Casida JE (1997). Anomalous Structure-Activity Relationships of 13-$$homo$$-13-Oxarotenoids and 13-$$homo$$-13-Oxadehydrorotenoids. Chem. Res. Toxicol..

[33] Ueno H, Miyoshi H, Ebisui K, Iwamura H (1994). Comparison of the inhibitory action of natural rotenone and its stereoisomers with various NADH-ubiquinone reductases. Eur. J. Biochem..

[34] Büchi, G., Crombie, L., Godin, P. J., Kaltenbronn, J. S., Siddalingaiah, K. S., & Whiting, D. A. 553. The absolute configuration of rotenone. *J. Chem. Soc.* 2843–2860 (1961).

[35] Olsson MHM, Søndergaard CR, Rostkowski M, Jensen JH (2011). PROPKA3: Consistent Treatment of Internal and Surface Residues in Empirical pKa Predictions. J. Chem. Theory Comput..

[36] Bridges HR, Bill E, Hirst J (2012). Mossbauer Spectroscopy on Respiratory Complex I: The Iron-Sulfur Cluster Ensemble in the NADH-Reduced Enzyme Is Partially Oxidized. Biochemistry.

[37] Abraham MJ, Murtola T, Schulz R, Pall S, Smith JC, Hess B, Lindahl E (2015). GROMACS: High Performance Molecular Simulations Through Multi-Level Parallelism from Laptops to Supercomputers. SoftwareX.

[38] Darden T, York D, Pedersen L (1993). Particle mesh Ewald: An N$$\cdot$$log(N) method for Ewald sums in large systems. J. Chem. Phys..

[39] Huang J, Rauscher S, Nawrocki G, Ran T, Feig M, de Groot BL, Grubmuller H, MacKerell AD (2017). CHARMM36m: an improved force field for folded and intrinsically disordered proteins. Nat. Methods.

[40] Jorgensen WL, Chandrasekhar J, Madura JD, Impey RW, Klein ML (1983). Comparison of Simple Potential Functions for Simulating Liquid Water. J. Chem. Phys..

[41] Chang CH, Kim K (2009). Density Functional Theory Calculation of Bonding and Charge Parameters for Molecular Dynamics Studies on [FeFe] Hydrogenases. J. Chem. Theory Comput..

[42] McCullagh M, Voth GA (2013). Unraveling the Role of the Protein Environment for [FeFe]-Hydrogenase: A New Application of Coarse-graining. J. Phys. Chem. B.

[43] Galassi VV, Arantes GM (2015). Partition, Orientation and Mobility of Ubiquinones in a Lipid Bilayer. Biochim. Biophys. Acta.

[44] Teixeira MH, Arantes GM (2019). Effects of Lipid Composition on Membrane Distribution and Permeability of Natural Quinones. RSC Adv..

[45] Best, R. B. *et al.* Optimization of the Additive CHARMM All-Atom Protein Force Field Targeting Improved Sampling of the Backbone $$\phi$$, $$\psi$$ and Side-Chain $$\chi$$1 and $$\chi$$2 Dihedral Angles. *J. Chem. Theory Comput.* **8**, 3257–3273 (2012). 10.1021/ct300400x PMC3549273 23341755

[46] Lee C, Yang W, Parr R (1988). Development of the Colle-Salvetti Correlation-Energy Formula into a Functional of the Electron Density. Phys. Rev. B.

[47] Becke, A. D. Density Functional Thermochemistry. III. The Role of Exact Exchange. *J. Chem. Phys.* **98**, 5648 (1993).

[48] Ditchfield, R., Hehre, W., Pople, J. A. Self-Consistent Molecular-Orbital Methods. IX. An Extended Gaussian-Type Basis for Molecular-Orbital Studies of Organic Molecules. *J. Chem. Phys.* **54**, 724–728 (1971).

[49] Pereira, C. S., Teixeira, M. H., Russell, D. A., Hirst, J., & Arantes, G. M. Dataset: Mechanism of rotenone binding to respiratory complex I depends on ligand flexibility (2023). 10.5281/zenodo.7781922. PMC10130173 37185607

[50] Barducci A, Bussi G (2008). Parrinello, M.

[51] Tribello GA, Bonomi M, Branduardi D, Camilloni C, Bussi G (2014). Plumed 2: New feathers for an old bird. Comput. Phys. Commun..

[52] Roux B (1995). The Calculation of the Potential of Mean Force Using Computer Simulations. Comp. Phys. Comm..

[53] Serreli, R. Pharmacological aspects of the inhibition of mammalian respiratory complex I. Ph.D. thesis, University of Cambridge, United Kingdom (2018).

[54] Klopman G, Li J-Y, Wang S, Dimayuga M (1994). Computer automated log P calculations based on an extended group contribution approach. J. Chem. Inf. Comput. Sci..

[55] Wang P, Dhananjayan N, Hagras MA, Stuchebrukhov AA (2021). Respiratory complex I: Bottleneck at the entrance of quinone site requires conformational change for its opening. Biochim. Biophys. Acta.

[56] Dhananjayan, N., Wang, P., Leontyev, I., & Stuchebrukhov, A. A. Quinone binding in respiratory complex I: Going through the eye of a needle. The squeeze-in mechanism of passing the narrow entrance of the quinone site. *Photochem. Photobiol. Sci.* **21**, 1–12 (2021). 10.1007/s43630-021-00113-y PMC8799541 34813075

[57] Grivennikova VG, Maklashina EO, Gavrikova EV, Vinogradov AD (1997). Interaction of the mitochondrial NADH-ubiquinone reductase with rotenone as related to the enzyme active/inactive transition. Biochim. Biophys. Acta.

[58] Reis AAO, Sayegh RSR, Marana SR, Arantes GM (2020). Combining Free Energy Simulations and NMR Chemical-Shift Perturbation To Identify Transient Cation-$$\pi$$ Contacts in Proteins. J. Chem. Inf. Model..

[59] Åqvist J, Luzhkov VB, Brandsdal BO (2002). Ligand Binding Affinities from MD Simulations. Acc. Chem. Res..

[60] Nunes-Alves A, Arantes GM (2014). Ligand-receptor affinities computed by an adapted linear interaction model for continuum electrostatics and by protein conformational averaging. J. Chem. Inf. Model..

[61] Friedrich T, Van Heek P, Leif H, Ohnishi T, Forche E, Kunze B, Jansen R, Trowitzsch-Kienast W, Höfle G, Reichenbach H, Weiss H (1994). Two binding sites of inhibitors in NADH: ubiquinone oxidoreductase (complex I) Relationship of one site with the ubiquinone-binding site of bacterial glucose: ubiquinone oxidoreductase. Eur. J. Biochem..

[62] Ueno H, Miyoshi H, Inoue M, Niidome Y, Iwamura H (1996). Structural factors of rotenone required for inhibition of various NADH-ubiquinone oxidoreductases. Biochim. Biophys. Acta.

[63] Fang J, Wang Y, Beattie DS (2001). Isolation and characterization of complex I, rotenone-sensitive NADH: ubiquinone oxidoreductase, from the procyclic forms of Trypanosoma brucei. Eur. J. Biochem..

