# Supplementary material for: Mechanism of rotenone binding to respiratory complex I depends on ligand flexibility
Source: Sci Rep. 2023 Apr 25;13:6738. doi: 10.1038/s41598-023-33333-6 (PMC10130173; doi:10.1038/s41598-023-33333-6)
Supplement: Supplementary file 1 — Supplementary Information. [file 41598_2023_33333_MOESM1_ESM.pdf]

Supporting Information

Mechanism of rotenone binding to respiratory complex I depends on ligand flexibility

Caroline S. Pereira, Murilo H. Teixeira, David A. Russell, Judy Hirst* and Guilherme M. Arantes*

Department of Biochemistry, Instituto de Química, Universidade de São Paulo, Av. Prof. Lineu Prestes 748, 05508-900, São Paulo, SP, Brazil , MRC Mitochondrial Biology Unit, University of Cambridge, Cambridge Biomedical Campus, Hills Road, Cambridge, CB2 0XY, UK and Department of Chemistry, University of Cambridge, Lensfield Road, Cambridge, CB2 1EW, UK

Email: jh@mrc-mbu.cam.ac.uk ; garantes@iq.usp.br

Supplementary Methods

The simulation model was based on the cryoEM structure of complex I from *Mus musculus* obtained at a 3.1Å global resolution (PDB 6ZR2).[22] Protonation states of side-chains were equivalent to the free amino-acid protonation in neutral pH, except for His59^{NDUFS2}, His549^{NDUFS1} and His42^{NDUFB2} which were di-protonated (Hsp), and Glu68^{ND3}, Glu36^{NDUFS5}, Glu262^{ND1} and Glu114^{ND4} which were protonated. These alternative protonation states were chosen based on PropKa[35] calculations and analysis of the chemical environment of these side-chains (exposure to lipids and H-bonding). The N-termini of NDUFS7 (Ser34) and truncated NDUFB6 (Ser66) were set to neutral, as these are embedded in the membrane. Neutral His tautomers were chosen based on optimal H-bonding. As previously proposed,[36] the redox state of each FeS cluster along the redox chain was interleaved in reduced and oxidized forms, starting with the N2 cluster reduced. The total charge of the protein complex was -14 $|e|$. Retained phospholipids were built with linoleoyl (18:2) acyl chains. Missing hydrogen atoms were built with the GROMACS suite.[37]

The protein complex was inserted in a bilayer with composition mimicking the inner mitochondrial membrane.[44] The final system contains 368 DLPC (179 in the matrix leaflet), 294 DLPE (143 in the matrix leaflet), 96 CDL (di-anion, half in each leaflet) and 22 oxidised Q_{10} molecules. The asymmetry in DLPC and DLPE composition is due to chain NDUFA9 occupying an area of the matrix leaflet only. To neutralise the total charge and maintain a 0.1 M salt concentration, 553 Na^{+} and 348 Cl^{-} ions were included. The simulation model was relaxed and equilibrated during molecular dynamics (MD) simulations as described in the main text.

Supplementary Figures

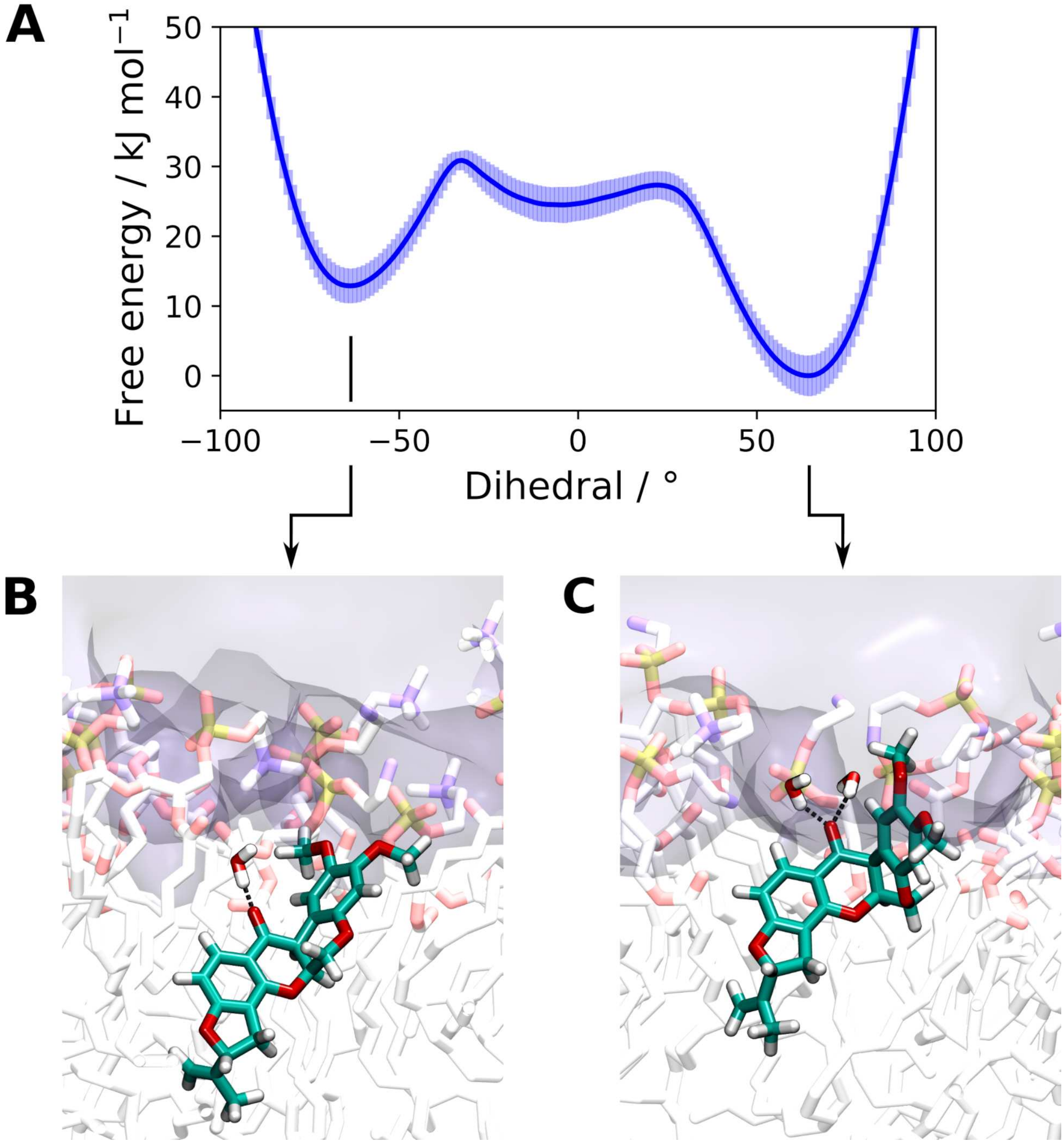

Figure S1: Rotenone conformer interconversion in mixed membrane (DLPC + DLPE + CDL + Q_{10}). (A) Free energy profile obtained from metadynamics simulations along the χ_2 dihedral. Stable rotenone position while embedded in the membrane for (B) straight and (C) bent conformations. Note the straight form localizes ~2 Å deeper in the membrane.

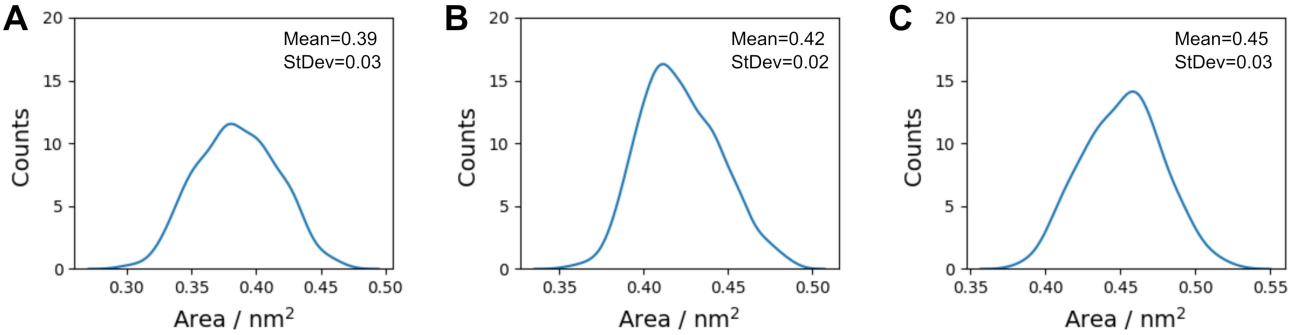

Figure S2: Area of the triangle determined by the C_α position of ND1 Ala18, Ala52 and Phe224 in the structure bottleneck at the Q-channel entrance (Fig. 1). This is a conservative estimate of the area available at the cross-section of the bottleneck for ligand passage and binding from the membrane in a closed conformation of the Q-channel.[12] This area is 0.40 nm^2 and 0.39 nm^2 in the original structure used for our model (PDB 6ZR2) and in bovine complex I bound to Q_{10} (PDB 7QSK),[15] respectively. Panels show the normalized distribution of this area obtained from 170 ns of MD simulations when rotenone is fully in the membrane (A, RC1= -0.3 nm, unbound Q-channel), and bound in the bottleneck (RC1=0.8 nm) in straight (B) and in bent (C) conformations. Mean and standard deviations (StDev) are also shown. The estimated transversal area of rotenone in straight form is $\sim$0.3 nm^2 (= 0.75 nm $\times$ 0.4 nm, respectively the lenght and the thickness considering van der Walls radii of rotenone D-E rings, which are placed at the bottleneck in RC1=0.8 nm). Thus, there is enough area in the bottleneck for passage of straight rotenone. The increase in mean area when rotenone binds to the bottleneck in straight form (0.03 nm^2 = 0.42 - 0.39 nm^2) is similar to the thermal fluctuation of the area in the unbound Q-channel (A, StDev = 0.03 nm^2) and does not represent a significant free energy cost for binding. For the bent conformer, however, the mean area increases twice as much (0.06 nm^2 = 0.45 - 0.39 nm^2), requiring deformation of ND1 helices in the bottleneck beyond their normal fluctuation. This deformation contributes to the higher free energy barrier found for passage in the bent form (Fig. 1F).

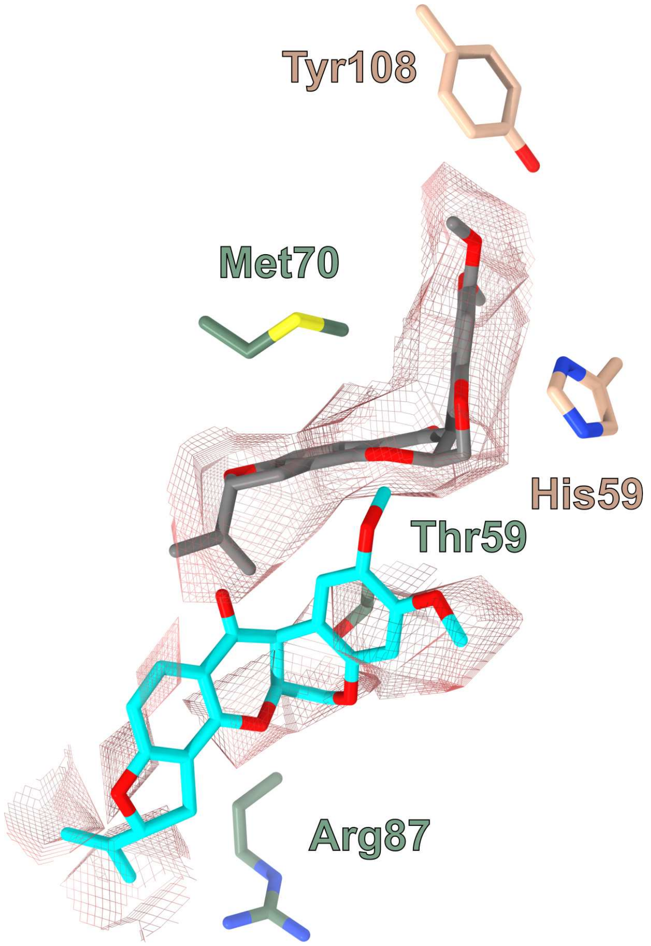

Figure S3: CryoEM density observed inside the Q-channel for the closed state map of *Sus scrofa* complex I preparation with NADH and rotenone (EMD-31651).[9] Positions obtained from simulations for rotenone in ROT1 and pre-redox modes are shown respectively in gray and cyan, respectively. Key residues are shown with names colored as their subunits in Fig. 1. The density inside the Q-channel, plotted as a red mesh using UCSF ChimeraX with threshold level 0.008, can approximately fit two rotenone molecules and can not be assigned to the protein chain.

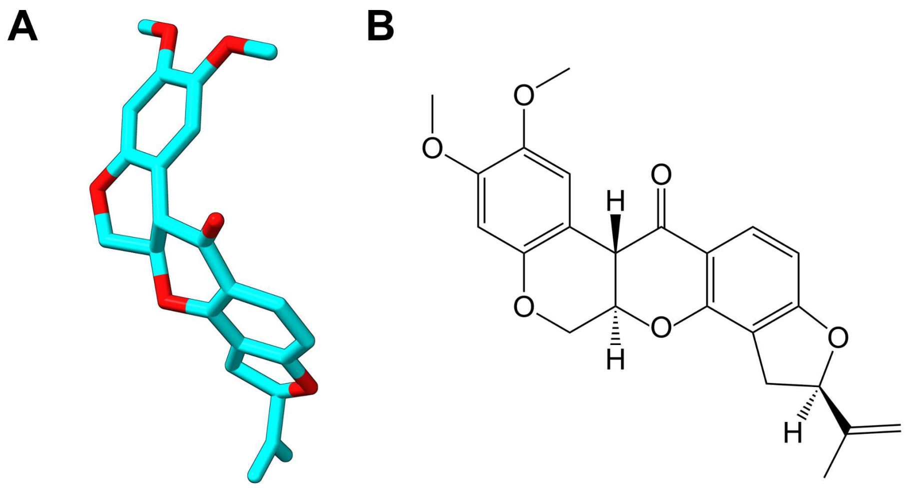

Figure S4: Rotenone epimer bound in the ND4 subunit (ROT3 mode) in the (A) cryoEM model (PDB 6ZKM)[8] and corresponding (B) chemical structure.

Table S1: Force-field parameters calibrated for torsion around bonds C1–C6 and C6–C5 of rotenone. Only parameters modified from the standard CGenFF set[45] are shown. Amplitudes for $n = 3, 4$ are zero.

**Dihedral**	**Phase (δ_n / °)**	**Amplitude ($\mathbf{K}_n$ / kJ mol^{-1})**	**Multiplicity (n)**
C12-C1-C6-C5	48.630	7.481	1
C12-C1-C6-C5	74.982	5.517	2
C12-C1-C6-HC6	89.358	5.594	1
C12-C1-C6-HC6	42.559	11.166	2
C2-C1-C6-C5	119.187	7.014	1
C2-C1-C6-C5	116.106	8.203	2
C2-C1-C6-HC6	112.635	7.913	1
C2-C1-C6-HC6	96.301	8.729	2
O11-C6-C1-HC1	52.794	14.510	1
O11-C6-C1-HC1	159.907	9.415	2
C1-C6-C5-O4	41.564	6.923	1
C1-C6-C5-O4	66.274	4.384	2
O11-C6-C5-O4	107.441	12.475	1
O11-C6-C5-O4	40.747	10.269	2
O11-C6-C5-HC5	122.544	6.099	1
O11-C6-C5-HC5	82.588	9.053	2

Table S2: Force-field parameters calibrated for torsion around bond C6–C5 of dehydrated rotenone. Only parameters modified from the standard CGenFF set[45] are shown.

**Dihedral**	**Phase (δ_n / °)**	**Amplitude ($\mathbf{K}_n$ / kJ mol^{-1})**	**Multiplicity (n)**
O11-C6-C5-O4	107.441	12.475	1
O11-C6-C5-O4	40.747	10.269	2
O11-C6-C5-HC5	122.544	6.099	1
O11-C6-C5-HC5	82.588	9.053	2
